# Recycled Aggregates Produced from Construction and Demolition Waste for Structural Concrete: Constituents, Properties and Production

**DOI:** 10.3390/ma14195748

**Published:** 2021-10-01

**Authors:** João Pacheco, Jorge de Brito

**Affiliations:** 1 CERIS, c5Lab: Sustainable Construction Materials Association, 2795-242 Linda-a-Velha, Portugal; jpacheco@c5lab.pt; 2 CERIS, IST, Universidade de Lisboa, 1049-001 Lisbon, Portugal

**Keywords:** coarse recycled aggregates, construction and demolition waste, production of aggregates, recycled aggregate concrete, circular economy

## Abstract

This paper concerns the recovery of construction and demolition waste as coarse recycled aggregates for concrete. Coarse recycled aggregates may be used as a partial or total replacement of natural aggregates, contributing to the circular economy and minimizing landfill disposals as well as the consumption of natural mineral resources. However, construction and demolition waste is a heterogeneous material with undefined quality and the processing of this waste into recycled aggregates needs to ensure that the recycled aggregates have suitable properties for concrete. This paper summarizes several aspects related to coarse recycled aggregates, specifically addressing: (i) the typical composition of construction and demolition waste; (ii) the influence of different types of constituents on the properties of recycled aggregates and recycled aggregate concrete; (iii) requirements for recycled aggregates to be used in concrete; and (iv) production methods of recycled aggregates. It is argued that coarse recycled aggregates are a suitable construction material with adequate quality, even when common equipment is used in their production and preliminary separation as a key operation for ensuring the quality of the aggregates is recommended.

## 1. Introduction

Legislation is pushing the concrete industry towards environment-friendly options, including the recovery of construction and demolition waste (CDW) as a secondary raw material. The replacement of coarse natural aggregates (NA) with coarse recycled aggregates (CRA) sourced from CDW in the production of concrete is one such case that minimizes the consumption of natural mineral resources and provides a way to recover CDW as a raw material. The recovery of CDW is a pressing concern of the European Union (EU) that is being enforced through EU Directives, e.g., EU Directives 2008/98/EC [1] and 2018/851 [2], and Communications of the European Commission, e.g., the section on construction of the Circular Economy Action Plan [3]. Moreover, research worldwide shows that concrete with suitable properties may be produced with CRA. Nevertheless, industrial agents in most regions do not readily adopt recycled aggregate concrete because:

- Under given circumstances, NA may be less costly than CRA;
- Clients, designers, and contractors are still skeptical about CRA, a material seen as unreliable;
- In many regions, CDW plants do not produce CRA suitable for concrete due to the absence of market.

Because of these hindrances, the market uptake of recycled aggregates (RA) by the concrete industry is quite limited. As presented in the 2019–2020 annual review of UEPG (the European Aggregates Association) [4], only 10.6% of all aggregates produced in the EU + EFTA (the European Free Trade Association) were RA, most of which were CRA. Notwithstanding a relevant increase from the 6.1% figure of the 2017–2018 annual review [5], the fact is that the potential for increased uptake is large, since relevant country-to-country differences are observed: France (27%), the United Kingdom (26%), Netherlands and Malta (24%) and Belgium (21%) use a far larger amount of RA than the average. This shows that policy towards enforcement/encouraging the use of RA can significantly increase market uptake. Such measures are expected to occur in the future and construction agents and CDW plants need to be prepared for them.

An example of a measure that is already changing how the construction industry is using RA is EU Directive 2018/851 [2], which amended EU Directive 2008/98/EC [1]. EU Directive 2008/98/EC [1] stipulated a target of 70% of non-hazardous CDW reuse/recycling by the year 2020. However, this target was met in the majority of the EU with significant contribution of backfilling [6], which not only constitutes downcycling and is unintended [6,7] but may also, in fact, be landfilling under some conditions [8]. Therefore, EU Directive 2018/851 [2] limits backfilling operations to the *minimum necessary*. To understand the impact of this measure, in the year of 2016, backfilling amounted to 50% of the mineral fraction of recovered CDW in Portugal [6].

This implies that uses for RA other than downcycling are needed. Using CRA in concrete is one such type of recovery of CDW and would contribute meaningfully to the circular economy since concrete is made of up to 80% of aggregates by volume and is the second most used material after water. Moreover, the access to NA in some regions is becoming scarce [9] and alternative sources of aggregates are required.

## 2. Generation of Construction and Demolition Waste

Legislative steps towards CDW recovery are a natural consequence of the overwhelming yearly generation of CDW. According to Eurostat, CDW consistently amounts to over 35% of all waste produced in the EU, with a yearly generation of about 1000 million metric tonnes, or 2.2 metric tonnes per capita. Due to the nature of CDW, which result from construction, retrofitting and demolition activities, their composition is variable [10,11,12].

The characterization of CDW by type is very relevant since CRA for concrete should be mostly composed of stone and concrete waste, followed by ceramics [13]. Other constituents of CRA have deleterious influence on concrete properties and should be avoided. These constituents include glass, plastics, metals, clay, bituminous materials and gypsum-based materials. These types of constituents have different unintended effects on concrete, such as changes in setting time, weak bond between CRA and the cement paste, damaging chemical reactions, and worse fresh-state and hardened-state properties of concrete.

Table 1 presents the composition by weight of CDW from several EU countries as presented in a report [8] commissioned by the EU. This table defines the types of waste in terms of the European List of Wastes and excludes soils.

As seen in Table 1, notwithstanding the majority of CDW consisting of concrete and ceramics, significant contents of other materials are still present. This means that preliminary sorting and removal of unintended types of waste is required (as discussed in Section 4). Moreover, the reported composition of CDW differs relevantly from country to country. This is due to several aspects, such as construction tradition of a region, labour costs, equipment, economic context and legislation on waste management, including the criteria used to classify waste. Nevertheless, these results agree with those reported for other regions, such as Australia [14] and the People’s Republic of China [12,15].

CDW is sent to licensed CDW plants where waste is separated by type. The fraction that is used to produce RA then undergoes a processing stage characterized by removal of deleterious materials, crushing and size classification, ultimately resulting in RA that may be used for different types of applications, such as backfilling and road construction (these uses are seen as downcycling) and CRA for concrete, which is the topic of this paper. Section 4 deals with how CDW is processed into CRA fit for concrete production.

## 3. Coarse Recycled Aggregates

### 3.1. Constituents of Coarse Recycled Aggregates

CRA are composed of several constituents and this is a natural consequence of the different types of waste present in CDW (Table 1). The European Standard EN 933-11 [16] defines the following constituents of CRA:

- R_u_—unbound stone (in fact, natural aggregates);
- R_c_—concrete and mortar;
- R_b_—clay masonry, calcium-silicate masonry, aerated non-floating concrete;
- R_a_—bituminous materials;
- R_g_—glass;
- X—other materials (clay, soils, metals, non-floating wood, plastic, gypsum-based and rubber);
- FL—floating materials.

These constituents have different properties and their suitability as aggregates for concrete are not the same. As found in [13], the smaller the water absorption and the larger the density, the more adequate a CRA is for concrete. R_u_ (which is, in practical terms, an NA) is the most adequate constituent, followed by R_c_ and R_b_. All other constituents should be seen with caution and standards and national specifications limit the incorporation of CRA based on the contents of constituents (see Section 3.3). As a general rule, CRA should have as much constituents of the type R_u_ and R_c_ as possible.

This is also the main reason why the use of CRA is preferred in detriment of fine recycled aggregates (FRA), since FRA are composed of constituents that are unintended, e.g., contents of the type X and disaggregated mortar (a specific poor-quality constituent present in types R_c_ and R_b_). This means that when CRA are compared with FRA of the same source, significant differences are found and the CRA is a better aggregate [17,18]. Therefore, standards and recommendations are more stringent in what concerns the use of FRA than in what concerns the use of CRA.

To ensure that the CRA are of sufficient quality to be used in concrete, the production process includes preliminary separation and RA may be produced from two types of CDW:

- Mixed CDW, which is achieved by removal of most unintended materials (e.g., wood, large plastics, soils);
- Concrete waste, since this type of waste is of good quality (mainly constituents of the type R_u_ and R_c_, with a small content of contaminants since preliminary sorting is not perfect).

Based on their results, the authors of [19] argued that the production of good-quality CRA for concrete requires that the content of wood, plastic, glass and asphalt waste is as low as possible. This is achieved through preliminary sorting and removal of deleterious materials during the production of the CRA. The typical composition of CRA produced with mixed CDW is as follows [20,21,22]:

- Content of R_c_ plus R_u_ of about 65% to 85%;
- Content of R_b_ in the region of 10% to 35%;
- Content of R_g_ and R_a_ between 0% and 2%, but in specific cases of up to 10%;
- Content of X below 2%.

When CRA are produced from concrete waste, the typical content of R_c_ + R_u_ is larger than 90% and the remaining CRA are mainly composed of R_b_ [23].

Since the processing described in Section 4 removes unintended constituents, the contents of R_c_ and R_u_ are larger than what would be expected by analyzing the contents shown in Table 1 for each type of CDW. Conversely, the contents of some types of CDW, namely, those that have commercial value (metals) and those that impair the properties of concrete the most (e.g., gypsum-based), are greatly reduced. This is relevant because:

- Most properties of concrete are detrimentally affected by ceramics [24]. Ceramics are porous and weak and their presence decreases the aggregate crushing value of the CRA [25] and results in larger number of transaggregate fractures in the mechanical failure mechanisms of concrete [18].
- Clay has different detrimental effects. Fine particles of clay may cover the particles of CRA, weakening the bond between the aggregate and the cement paste. Furthermore, since these particles are smaller than those of cement, they may also adsorb to the cement particles, impairing a regular and homogeneous crystallization of the cement hydrates [26]. Other detrimental effects are due to their large water absorption, which may compromise workability if unaccounted for, and the possible influence on the setting and hardening of concrete. Clay may be present as agglomerated lumps of relatively large dimension (including within the coarse aggregate size range), especially when moist. These large clay particles tend to disaggregate during handling, transport and mixing.
- Gypsum-based materials, including plasters, may induce sulphate reactions that influence setting [17] and, most importantly, these materials can lead to sulphate attack of hardened concrete, resulting in expansion, cracking and spalling [26].
- Glass and plastics bond poorly with the binder and metallic constituents are prone to corrosion. These types of constituent are typically poorly shaped for concrete (too flaky and/or elongated).

Apart from these considerations, aggregates should be stiff, relatively round, have small porosity and be relatively strong [27,28].

The nanoindentation tests of [19] are particularly useful to understand the mechanical properties of specific constituents of the CRA and the respective interfacial transition zone (ITZ) between aggregates and binder paste: these authors produced concrete made with CRA sourced from mixed CDW, and the nanoindentation tests presented in Table 2 show that the type of constituent is a determining factor for its stiffness, as well as that of the ITZ. The results of glass constituents are particularly interesting: they are much stiffer than all others, yet their poor bond with the binder paste leads to a porous and deformable ITZ.

### 3.2. Properties of Coarse Recycled Aggregates

The specificities of the constituents of CRA mean that, in comparison to NA (raw stone), CRA are weaker [29], more deformable [30], more porous and lighter [31] and have larger water absorption [32]. Because of their composition, CRA are also rougher than NA [33]. In terms of geometry, CRA tend to be flakier and more elongated, which is mostly due to two reasons:

- The nature of part of their constituents (e.g., those of type R_b_, such as ceramic tiles and bricks), which tend to fragment into elongated shapes;
- As seen in Section 4, many CDW plants use primary crushing only, typically with a jaw crusher. This will also result in more elongated particles [34].

Table 3 gives the mean 24 h water absorption and saturated surface-dry density of three types of RA: concrete waste (constituents of type R_c_ and R_u_); masonry and concrete waste (constituents of type R_c_, R_u_ and R_b_); and unsorted CDW waste. These data were compiled by a metanalysis that covers hundreds of RA tests [13]. The same source argued for a classification system that defines the suitability of RA based on their density and water absorption.

When these data are compared, it is clear that CRA produced from concrete waste are those with the best properties for concrete (smaller water absorption and larger density). Moreover, FRA have worse properties than CRA. When the water absorption of CRA is compared with that of coarse crushed limestone, which is in the range of 1.0% to 2.5%, it is clear that the water absorption of CRA is much larger.

The large water absorption of CRA and FRA (Table 3) is mainly caused by their porosity and implies that the batching of concrete at ready-mixed plants needs to be done with special considerations. In laboratories, production pace allows the water absorption and the water content of the RA to be properly measured and accounted for when defining the effective w/c ratio [35]. However, at industrial scale, neither the water absorption of the specific lot of RA currently at the plant nor the water content of the RA may be measured without compromising production time. Moreover, [36,37] found that the fast experimental methods used in ready-mixed plants to determine the water content of aggregates are not adequate for RA since they only accurately measure the content of water at the surface of the aggregate. This may have relevant influence on the effective water content of recycled aggregate concrete mixes and may lead to relevant decreases of mechanical properties [32]. Due to these considerations, preference should go to the used RA at pre-saturated moisture conditions.

Limestone NA have a saturated-surface-dry density between 2600 kg/m^3^ and 2700 kg/m^3^. The smaller density of CRA needs to be accounted for in mix design, since if NA are directly replaced with CRA based solely on weight, the volume of aggregates becomes larger, at the expense of smaller content of binder paste.

A specific problem of CRA is their variability [10,29,38], since the different batches of CRA provided to a concrete producer depend on the quality of the original CDW waste. This variability is relevant even if the constituents of CRA are of R_c_+R_u_: different concrete (waste) compositions have different mix design and the type of NA in a concrete (waste) composition used is not necessarily the same. This is understood in [39], where a large lot-to-lot variability of the 24 h water absorption (between 4.9% and 11.9%) of CRA produced from concrete waste was found.

Because the typical production process of CRA is not as optimized as that used to produce NA (namely, in what concerns crushing and sieving), the grading of CRA may not be as suitable for concrete as that of NA and the voids content of CRA tends to be larger, with detrimental influence on the mechanical and durability properties of concrete [37].

The chemical contamination of CRA is not typically discussed but is a relevant aspect. CDW arrives at the CDW plant from several sources and different types of contaminants may be present and impair the properties of concrete. The influence of gypsum- and clay-based constituents has already been mentioned. CRA may be contaminated with organic matter and salts (chlorides, sulphates and others) from several origins and, depending on the substance, the detrimental effects range from changes in setting and hardening of concrete, long-term adverse chemical reactions (such as sulphate attack [17]), efflorescence, and reinforcement corrosion. The overall content of sulphates of CRA may be large since they are the sum of the sulphates of both the adhered mortar and NA of the concrete waste.

### 3.3. Requirements of Coarse Recycled Aggregates for Concrete

This section presents requirements for the use of CRA in concrete in regions that follow the European Standards (EN). As stated in [37], a similar framework is used in other places and regional differences are mostly due to previous experience in the use of CRA.

Most of the requirements for CRA are the same that NA must comply with. The majority of them are found in EN12620 [40], where properties, requirements and classification of aggregates for concrete are presented, and EN206, Annex E [41], where recommendations for some of the properties of CRA presented in EN12620 [40] are given. Both documents also include clauses to address specific doubts about the characteristics of CRA. Most standards that include clauses for CRA and recycled aggregate concrete state recommendations for their use instead of enforcing strict conditions. This is partly due to CRA being a variable material.

The requirements presented in this section concern steel-reinforced concrete, since this is by far the most produced type of structural concrete. EN206 [41] also makes allowance for specific national documentation concerning the use of CRA in concrete, such as the Portuguese Specification LNEC E471 [42].

Concerning grading, the general requirements of EN12620 [40] for NA apply. However, the fines content of CRA is typically limited (e.g., in LNEC E471 [42] to either 3% or 4%, depending on the constituents of CRA, while Annex 15 of the Spanish EHE 08 [43] limits fines to 1.5%). This may be difficult to achieve due to the roughness, friability and small particles attached to the constituents of CRA.

The same shape classes of NA are in use for CRA. In Annex E of EN206 [41], the classes FI50 (maximum 50% of flaky particles by weight) and SI55 (maximum 55% of elongated particles by weight) are recommended.

The resistance to fragmentation of coarse aggregates is determined through the Los Angeles coefficient. Annex E of EN206 [41] recommends a maximum Los Angeles coefficient of 50 for NA and CRA alike. However, Paine and Dhir [24] tested the incorporation of several CRAs in concrete and argue that the Los Angeles coefficient of CRA should be at most 40, since they found that the influence of a CRA on the mechanical properties of common-strength concrete is correlated to the Los Angeles coefficient and CRA with larger LA coefficients are associated with relevant decreases of the w/c ratio in order to ensure equivalent mechanical properties. Decreases of w/c are unintended for economic and environmental reasons.

The requirement for volume stability of NA and CRA is also the same. Testing should follow EN1367-4 [44] and the tested shrinkage is restricted to 0.075% for NA and CRA alike. Due to the smaller stiffness of CRA, this may not be met if the content of poor constituents is too large. However, a large experimental campaign covering CRA from several origins [45] was reported that all CRA tested (out of 10) complied with this condition.

EN12620 [40] also states requirements for chemical contamination. Some of these are specific to certain origins of aggregates (e.g., shell content) and are not presented here. The standard has specific clauses for CRA, related both to test procedure and to requirements.

Chloride content should not be tested as typically done for NA (water-soluble chloride content), but for acid-soluble content. Nevertheless, EN12620 [40] states that the latter test overestimates the content available for chemical reactions. No recommended or required test values are stated and this test result should be declared whenever demanded by the client.

EN206 [41] recommends that the content of acid-soluble sulphates be limited to 0.8% by mass. No specific mention to CRA is made. In the case of LNEC E471 [42], the same limitation is specifically imposed for CRA. Annex E of EN206 [41] recommends that the content of water-soluble sulphates be restricted to 0.2% by mass. In [46], it is argued that, despite no experimental evidence of increased sulphate content due to constituents of the type R_b_, some bricks are produced with clay that is rich in sulphates. Documents that investigated several FRAs and CRAs state that sulphate contents are typically complied with by RA, except in rare instances [18,45].

RA should also comply with the requirements for alkali–silica reactivity, which are country-dependent and not defined in the EN set of standards. Since glass waste is typically removed before demolition, glass constituents are not present in sufficient content to lead to relevant reactivity. However, alkali–silica reactions are an important concern of CRA [17] because the attached mortar of R_c_ constituents is potentially rich in alkalis and the stone present in R_c_ and R_u_ constituents may be potentially reactive [47]. Nevertheless, investigations on this topic show that CRA are not expected to lead to alkali–silica reactions [46] unless CDW includes a relevant portion of pavement waste [42,48]. It should be noted that some authors have questioned the suitability of the conventional tests used for NA when assessing the reactivity of CRA [49].

The influence of constituents that affect setting and hardening of concrete must be checked according to EN12620 [40]. In there, organic matter should be checked through the humus content and, whenever there is a relevant content of humus, fulvic acid testing should follow. Both test protocols are defined in EN1744-1 [50]. If humus and fulvic acid contents are large or whenever sugar contents may be relevant (the content of sugars is not captured by either of the aforementioned tests), the influence on setting and hardening should be checked on mortar specimens. For this purpose., the aggregates need to be crushed to a specific fine grading and the limits for the increase in stiffening time and decrease of the 28-day compressive strength of the mortar tests stated in EN12620 [40] should be respected. In the specific case of CRA, EN12620 [40] includes testing of the influence of water-soluble materials—in this case, the test should follow EN1744-6 [51] and Annex E of EN206 recommends that the corresponding test result (delay in setting time) does not exceed 40 min.

In some regions, leaching requirements are also imposed [42]. An extensive study [46] found that the use of CRA increases potential for the leaching of sodium, potassium and chloride ions. However, the leaching of calcium decreases and no evidence for increased leaching of heavy metals is reported.

Compliance with most of the requirements presented in this section depends, as a first approximation, on the constituents of the CRA—e.g., the content of water-soluble sulphates is mostly due to gypsum/plasterworks [13]. Testing for constituents is made by mass and according to EN933-11 [16]. EN12620 [40] defines classes for the constituents of CRA (e.g., CRA of class R_c_90 must have at least 90% of constituents of the type R_c_). Since constituents of the types R_b_, Ra, X and FL should be present in the lowest amount possible, the designation of their class gives the maximum amount of the constituent (e.g., CRA of class R_b_30 have a maximum content of R_b_ equal to 30%. Based on the classes of each constituent, Annex E of EN206 [41] presents two types of CRA and defines maximum incorporation ratios (by weight) based on the environmental exposure class (Table 4). The standard specifies that the table assumes that CRA concern fractions above 4 mm only.

## 4. Processing of CDW into Recycled Aggregates

The quality of a CRA depends on the equipment and processes used to produce it [52]. Several combinations of equipment and processes are available and this section summarizes processes and their relevance. Apart from the need to remove unintended waste, the production of CRA is not largely different from that of NA: bulky materials are transported, crushed and screened by size. Moreover, the production has to ensure that CRA are of good quality and comply with the requirements presented in the previous section of this paper.

CDW is delivered to a licensed CDW plant by truck and an initial inspection and acceptance of the load is made. This inspection checks whether CDW complies with the composition declared to the CDW plant before accepting the load. The composition of CDW delivered depends on the efforts spent at the construction/demolition site for the separation of different types of waste. To promote separation by the contractor, the delivery of loads of mixed CDW is more costly than the delivery of separated types of CDW. Moreover, as stated in [46], contractors whose loads do not conform with the declared type of CDW and/or legislative requirements may be banned from the CDW plant.

After acceptance of the load, CDW either:

- Is immediately sent for processing into RA, whenever CDW is delivered with low contamination of unintended constituents; or
- Undergoes preliminary removal of unintended constituents and sorting whenever a significant portion of such wastes is included. At this stage, constituents that should not be included in RA are removed, e.g., wood, plasterboards, plastics, asphalt. This operation is usually made with an excavator.

In either case, large CDW blocks are fragmented with a hydraulic hammer or a hydraulic clamp in order to have adequate size and weight for processing.

**Figure 1:**
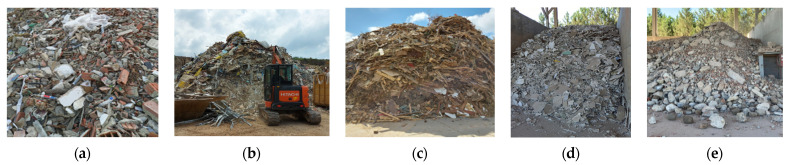
Separation of CDW by type(**a**) CDW requiring separation (**b**) Ongoing separation (**c**) Wood waste (**d**) Plasterboard waste (**e**) CDW—suitable.

Figure 1 shows different types of waste. Only the fraction labelled as “CDW—suitable” is used to produce RA. This may be either mixed CDW (as shown in Figure 1) or only concrete waste. The removal of unintended constituents is fundamental in order to ensure that the RA behave satisfactorily—see Section 3.1 and Section 3.3.

At the feeder of the processing line and before crushing, the CDW plant may:

- Include a bar screen that removes unintended types of waste that passed the preliminary separation (e.g., plastics);
- Sieve and remove the finer fractions of materials. This serves a twofold purpose: excess of fines reduces crushing performance [46] and the smaller fractions of CDW are mainly composed of deleterious materials (e.g., clay, soils, plastics, paper—see Section 3.1).

**Figure 2:**
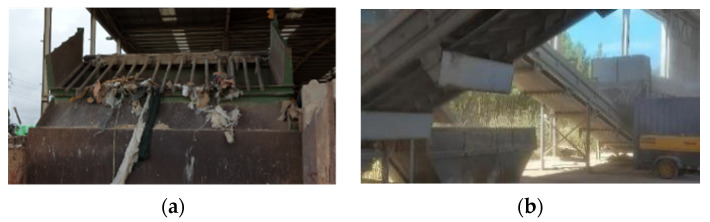
Initial sieving at the feeder (**a**) Removal of large and elongated materials with bar sieve (**b**) Removal of finer materials.

Figure 2 shows both types of initial sieving. The removal of finer materials is quite common.

After the initial screening, CDW is transported for crushing. Transport is generally made by conveyor belts throughout the production process of CDW plants.

The most used types of crusher are jaw crushers, impact crushers and cone crushers [26]. Jaw crushers are made of two plates, one of them stationary. As the other plate swings back and forth, particles are crushed between the plates and they exit the crusher when their size is equal or below the opening of the crusher. Jaw crushers are particularly suitable for primary crushing of CDW since they can be fed with waste of large size (and reinforcement) without clogging [52]. This is the same reason why jaw crushers are popular options in quarries for the primary crushing of NA. However, the shape of aggregates produced with jaw crushers tends to be elongated [26].

Impact crushers are suitable crushers for primary crushing. This type of crusher has a rotor that throws particles into steel plates, breaking them. Particles are kept inside the crusher and thrown into the steel plates until their size is smaller than the openings of the exit of the crusher. This type of crusher produces aggregates with good shape [26].

Cone crushers produce aggregates with high quality shape. The material is crushed between two cones: an internal fixed cone and an external cone that gyrates eccentrically. As a disadvantage, this type of crusher does not accept large particles and is not suitable for large particle size reductions (e.g., reductions of particles from 14 mm to 10 mm are ideal) [26]. Therefore, cone crushers are used as secondary or tertiary crushers.

As understood from this description, the production of crushed aggregates typically combines a jaw or impact crusher as a primary crusher and then uses other equipment for secondary or secondary and tertiary crushing (such as a cone crusher). This results in well-graded and shaped aggregates. However, for the production of RA this may not be feasible. The main activity of CDW plants is not the production of RA and in many regions the market does not demand good quality RA (because of the contextual reasons presented in Section 1). Therefore, it is not uncommon for CDW plants to resort to primary crushing only. In this case, a single jaw or impact crusher is typically used.

Silva et al. [52] argued that two and three crushing stages should be preferred ahead of primary crushing due to the better overall quality of the particles [34,53] of CRA and to higher predictability of the grading curve. However, other authors [54] differ and support primary crushing only, since, as the number of crushing stages increases, less CRA is produced (hence, the recovery rate of CDW as a secondary raw material decreases). Depending on the quality of the CRA, types of crusher used and number of crushing stages, CRA may make up between 40% and 80% of the crushed CDW [12,54,55].

The literature [37,56] on the most suitable type of primary crushing for CRA production has found that impact crushers tend to produce better-quality CRA than jaw and cone crushers. In [56], it was specified that impact crushers lead to better shape, larger density and smaller water absorption, but larger content of fines. The impact crusher results in better properties because of its operating principle: since particles reduce their size by being thrown into steel plates, their weakest and more friable phases tend to detach from the CRA.

**Figure 3:**
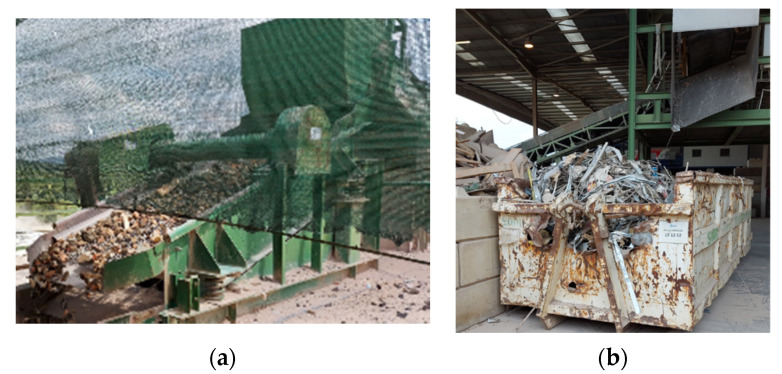
Crusher and magnetic separator (**a**) Impact crusher (**b**) Magnetic separator discharging ferrous metals removed during RA production.

Before and/or after crushing, magnetic separators remove ferrous metals. In other locations of the production line, additional magnetic separators may be used. The plant may also be equipped with Eddy current separators to remove non-ferrous metals. Figure 3 shows a crusher and a magnetic separator discharging ferrous metals during the production of CRA.

In addition, the preliminary separation is not sufficient to ensure that the constituents of CRA fully comply with the requirements of Table 4. Therefore, CDW plants resort to post-crushing removal of unintended constituents. Consequently:

- Lightweight materials are removed either with air sifting or wet separation. The former is characterized by strong air currents that separate materials by density, being efficient in the removal of materials with low density (e.g., glass and gypsum are not removed as efficiently as plastics since the density of the former is similar to that of constituents of types R_b_ and R_c_). Wet separation serves the same purpose and has the advantage of removing clay as well as chemical contaminants (e.g., water soluble chlorides and sulphates) through leaching [12,46].
- Hand sorting is made by visual inspections and manual removing of unintended wastes by operators. Typically, 3 to 6 operators perform this operation, therefore labour costs may be relevant. Hand sorting is usually made in designated areas and removed materials are sent directly to separated storage bins by gravity (usually the bins are below the sorting installation).
- Other more advanced technology may be used, such as automated sensor-based sorting [57]. Water or air jigs [57,58] may also be used to efficiently stratify the RA by density.

**Figure 4:**
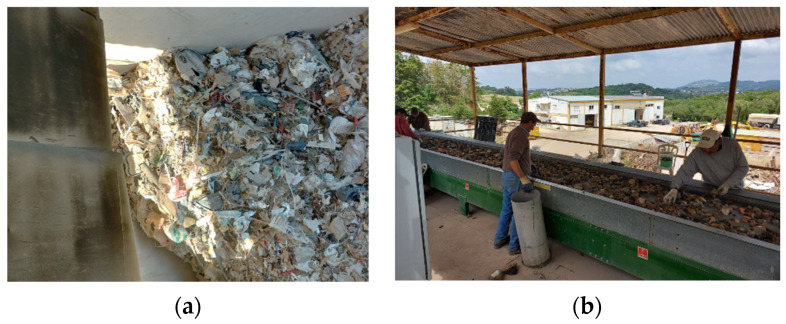
Traditional processes for the removal of unintended constituents (**a**) Lightweight materials removed by air sifting (**b**) Hand sorting.

Figure 4 shows lightweight materials removed by air sifting and operators performing hand sorting.

After the CDW is crushed into RA and the deleterious fractions are (mostly) removed, the final activity needed to produce CRA is the size classification of the RA. This is made by sieving, which can be made using different equipment, whose basic principle is the forced passage of particles below the sieve of a mesh through vibration.

**Figure 5:**
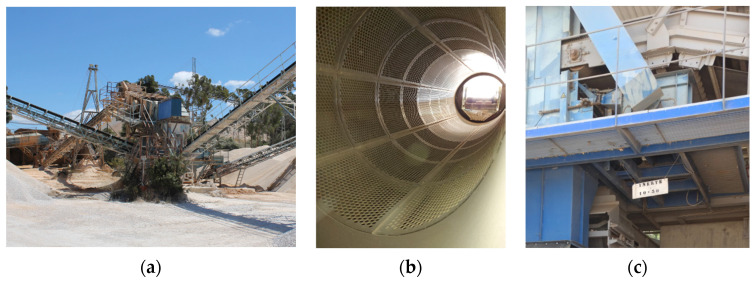
Crusher and magnetic separator (**a**) Stationary screener (**b**) Trommel (**c**) Perforated belt conveyor.

CDW plants deliver CRA of different size fractions and must use this type of equipment to separate the crushed particles by size. Most commonly, stationary screeners, in which sieving is made by vibrating an inclined deck where the mesh is installed, are used [26]. However, other equipment may be used, such as trommels (commonly used to produce aggregates for road construction) and perforated belt conveyors, which are compact solutions that save space. Figure 5 shows a stationary screener, a trommel and a vibration platform for a perforated belt conveyor.

After sieving, the aggregates are stored. Basic concerns related to the storage of CRA are care in handling and storage to minimize breakage [59], minimizing moisture due to the possible content of organic materials and unhydrated cement [52], and stockpiling with the intent to reduce heterogeneity [10,60], e.g., using a procedure similar to the “cell process” presented in [61].

The processes and equipment described in this section produce CRA of different quality, thus different CDW plants produce CRA of different quality. This occurs because some plants focus their business on the reception of CDW, others produce high-quality CRA, and others are adaptations of past quarries. When a CDW plant is an adapted quarry, crushing is typically of good quality and comprises up to three crushers. Primary crushing is made with a jaw or an impact crusher, while the crushing stages may be made with different equipment (e.g., hammer mill or cone crushers), size screening is made in stationary equipment that is prepared for the production of aggregates of different grading, and sorting and removal of contaminants depend on the investment made to adapt the quarry. CDW plants that are mostly focused on waste reception have simpler crushing (usually primary crushing, but it may be possible that CDW is not crushed and CRA are produced through size screening only) and this type of plant usually produces aggregates for road base applications. CDW plants that produce high-quality CRA use at least two crushers, well-performing sorting and removal methods (at least manual removal of large particles and air-based removal of light contaminants), and may wash the aggregates. Since the different production methods contribute to further material variability, compliance with requirements and certification are particularly relevant for the market acceptance of CRA. This section treated the most common technology and processes used to process CDW into RA. Other technology exists, such as mobile crushing stations [62], whose RA production process does not differ largely from that presented here. The major difference is that mobile plants are typically less equipped (e.g., single crushing is common and equipment for removal of unintended constituents is minimal) and CRA are of worse quality than those produced in fixed plants [63]. However, this statement is not consensual since some authors [64] argue that mobile crushers may be used to produce high-quality CRA from specific sources of concrete waste, since the concrete waste will be processed onsite without mixing with other CDW streams, as is presumed to be the case of fixed CDW plants.

Other technologically advanced processes for the production of CRA include equipment that optimizes production, such as technology that enables the optimized recovery of CRA, FRA and hydrated cement particles, e.g., electrodynamic fragmentation [65].

## 5. Discussion and Strategies to Promote Market Uptake of Recycled Aggregates

The previous section treated the common technology and processes used to process CDW into RA. More advanced technology exists but it is not used in the majority of regions [25] due to a current lack of relevant market demand for high quality CRA. This is expected to change, since the demand for CRA certified for concrete will increase [52] because of societal concerns, which drive the market towards Green-labelled products and lead to legislative measures that foster sustainable options by industrial agents [3].

Section 3.2 showed that, in general terms, the properties of CRA are not as suitable for structural concrete as those of NA. In addition, the CRA of different producers are of different quality, since production processes and equipment depend on the CDW plant. Therefore, the CDW plants should seek to ensure that their CRA consistently comply with standards and regulations because the market acceptance of CRA as an aggregate for concrete depends on the guarantee of a continuous supply of conforming CRA to the concrete industry.

In this context, beneficiation methods improve the quality of CRA and mitigate their heterogeneity and have been put forward as a solution for the compliance of CRA with standards. These beneficiating methods include [66,67,68]:

- Wrapping methods, in which a slurry is used to cover the particles of the CRA. This slurry may be made with different materials (e.g., cement, silica fume, fly ash) and will fill pores of the CRA, decreasing porosity and water absorption and increasing density, stiffness and strength. Alternatively, the surface of the CRA may be coated with water repellents [69].
- Thermal and acid treatments that remove most of the attached mortar from R_c_ constituents, either due to acid dissolution (e.g., using hydrochloric, sulfuric or acetic acid [70,71]) or through heating, which damages the hydrated products of the attached mortar of R_c_ constituents leading to their disaggregation.
- Mechanical removal of weaker/friable constituents of the CRA through abrasion [72]. This method is envisaged for CRA produced from concrete waste (constituents R_c_ and R_u_) and removes the attached mortar from R_c_ constituents. When the method is used for CRA with large content of weaker constituents (e.g., R_b_), a high amount of fines may be generated and the overall content of CRA may decrease drastically with excessive generation of recycled fines and FRA.
- Forced carbonation, which improves the properties of CRA through the reaction of carbon dioxide with the calcium substances present in the superficial attached mortar of R_c_ constituents. This reaction results in calcium carbonate and densifies the pore microstructure of the CRA [71,73].
- Ultrasonic cleaning, a method that removes loose fines from the CRA, improving bond between CRA and the new binder paste of concrete [66].

These treatments are mostly directed at reducing the detrimental effect of the attached mortar of R_c_ constituents and their beneficial effect has been experimentally demonstrated [70]. However, cost, environmental impacts and difficulties in industrial implementation are obstacles for the upscaling of beneficiation methods.

As a matter of fact, since partial incorporation ratios of CRA would be sufficient to take up all CDW generated yearly (in the case of the EU, an incorporation ratio of 20% would be sufficient [37]), partial incorporation of untreated CRA as an aggregate for concrete is a more practical and efficient way to valorize CDW in comparison to using beneficiated CRA at larger incorporation ratios.

In order to ensure that untreated CRA are of sufficient quality, the CDW used to produce them should be mainly composed of concrete waste. This ensures a high content of constituents R_b_ and R_u_ (see Section 3.1, Section 3.2 and Section 3.3). As shown in Section 4, the composition of CRA is controlled by preliminary separation of CDW and sorting of the CRA after crushing. Preliminary separation of CDW is a more reliable means of ensuring that the CRA has adequate composition since separation after crushing is not as efficient. Moreover, preliminary separation allows immediately sending the different types of waste (e.g., wood, glass and plastics) to their most suitable destiny, improving the overall efficiency of the recycling process and of waste recovery.

An alternative mean to ensure adequate composition is selective demolition [52], in which, prior to demolition, the construction is inspected and the waste sent to the CDW plants is better segregated. Moreover, selective demolition has other social and environmental advantages [74], such as a better handling and management of hazardous materials (such as asbestos) and improved valorization of elements of value that may be reused (such as ornamental stone, doors and windows). With selective demolition disposal costs are reduced but labour costs increase [74]—these changes in cost are not quantified in this paper because these figures depend on regional contexts [52,74]. Therefore, the economic viability of selective demolition is regional-dependent, and selective demolition has little economical appeal in many contexts, namely, when landfill taxes are small and the market value of recovered materials is low [13].

This paper is chiefly concerned with CRA, yet FRA and recycled fines are produced during the valorization of CDW as CRA and end goals for these products should be found. In this context, the following recovery options are promising prospects:

- The incorporation of FRA in 3D-printed concrete [75] due to the well-controlled conditions of 3D printing production (which allow a good characterization of the specific lot of FRA incorporated) and because the water absorption of the FRA may decrease printing time.
- The processing of recycled fines from concrete waste into recycled cement [76] due to the potential of thermoactivated cement to act as a hydraulic binder.
- The use of recycled sand in the production of clinker, because recycled sand is mainly made up of quartz and calcium carbonates. Successful industrial implementations of this concept are found in [37].

## 6. Conclusions

This paper presented the properties, requirements and production of coarse recycled aggregates produced from construction and demolition waste and meant for use in concrete. Coarse recycled aggregates are a viable material for the concrete industry. However, they are not the same as natural aggregates and their properties and compliance with standards are strongly dependent on the type of operations made both by the contractor (to separate the construction and demolition waste) and the construction and demolition waste plant.

The different production methods and the intrinsic heterogeneity of coarse recycled aggregates imply that they are a variable material and this results in reservations by the concrete industry. However, legislation is expected to change the current paradigm and coarse recycled aggregates will become a common raw material of the concrete industry. Therefore, construction and demolition waste plants need to produce coarse recycled aggregates that comply with requirements for use in concrete in a consistent way. These requirements seek to ensure that these aggregates are of good enough quality for their use in concrete, since, due to their nature, they are a worse material than conventional crushed natural aggregates and their use in concrete poses some concerns. The main concerns are solved by the requirements of standards and national specifications through:

- The classification of coarse recycled aggregates based on their constituents and the definition of minimum contents of intended constituents and maximum contents of unintended ones, so that construction and demolition plants ensure that unintended constituents are removed from the recycled aggregates during production.
- The classification by constituents is particularly relevant because of chemical contamination and due to the detrimental effect of clay on concrete. Washing would mitigate both problems, but is not common in construction and demolition waste plants. The compliance of the recycled aggregate with the content of constituents mitigates both effects, since clay content is reduced and constituents such as plasterboards and asphalt are removed to the extent possible, minimizing the potential for deleterious chemical reactions.
- Directly defining minimum demands for specific properties, including those related to mechanical behaviour (e.g., Los Angeles abrasion) and chemical contamination (e.g., the content of acid-soluble sulphates).
- Since water absorption and density are correlated with the overall quality of aggregates [13] and with industrial challenges for the production of recycled aggregates, some regulations opt to define maximum values for water absorption and minimum values for density.
- In addition, due to the variability of the properties of coarse recycled aggregates, it is common for standards to specify higher test frequencies for RA in comparison to NA.

The paper dealt with these issues and presented the common production method of coarse recycled aggregates, as well as beneficiation methods that allow the production of coarse recycled aggregates with better quality. However, due to industrial challenges and increased cost and environmental impacts associated to beneficiation, partial incorporation ratios of coarse recycled aggregates without beneficiation were argued for.

The paper also presented research trends on the use of other fractions of recycled materials (fine recycled aggregates, recycled fines and recycled cement).

Due to the very relevant differences in processing methods of recycled aggregates, construction and demolition waste plants may not be aware of the best possible methods that ensure that the recycled aggregate has sufficient quality to conform to the standards in use in a region. The clear definition of minimum best-practice demands for construction and demolition plants to produce recycled aggregates with suitable quality for structural concrete applications would be a very important step in this direction.

As future research, the authors recommend the following topics:

- Research on the recovery of other fractions of recycled aggregates. Fine recycled aggregates are typically studied for mortars but are not gaining industrial acceptance. Moreover, recycled fines also have no clear goal and research on the use of recycled cement, including the recovery process, is needed. Alternative uses for these materials are needed.
- The main motivation for the use of recycled aggregates is sustainability; therefore, the overall environmental impacts of the production of concrete with recycled aggregates should be smaller than those of concrete made solely with natural aggregates. This is not always the case and methodologies for the expedite estimation of the environmental impacts of both should be developed to aid decision-making.
- The performance of coarse recycled aggregates is still not fully understood in what concerns some phenomena, such as alkali–silica reactions and leaching. Better understanding of the conditions for coarse recycled aggregates to lead to unintended behaviour would be an important step to aid in future regulations.

## Figures and Tables

**Table 1:** CDW by type and country. Percentages by weight. Source [8], based on data from EUROSTAT.

Type of Waste	Croatia	Denmark	Estonia	Germany	Hungary	Luxemburg	Portugal	Slovakia
Concrete and ceramics	30%	56%	21%	59%	55%	69%	71%	36%
Wood, plastic and glass	1%	4%	6%	5%	1%	7%	3%	2%
Bituminous mixes	0%	0%	0%	20%	0%	0%	0%	0%
Metals	44%	15%	50%	9%	21%	11%	7%	45%
Insulating materials and materials with asbestos	2%	3%	1%	1%	6%	2%	1%	0%
Gypsum-based	0%	2%	1%	1%	5%	1%	0%	0%
Other CDW	22%	20%	21%	4%	21%	10%	17%	17%

**Table 2:** Results of nanoindentation tests on constituents of CRA, including the ITZ [19].

Constituent	Property	Value
R_c_	Mean Young’s modulus (aggregate)—GPa	20.7
	Mean Young’s modulus (ITZ)—GPa	25.4
	Minimum Young’s modulus (ITZ)—GPa	22.5
	Mean thickness (ITZ)—μm	55
R_b_	Mean Young’s modulus (aggregate)—GPa	32.5
	Mean Young’s modulus (ITZ)—GPa	22.9
	Minimum Young’s modulus (ITZ)—GPa	19.3
	Mean thickness (ITZ)—μm	40
R_a_	Mean Young’s modulus (aggregate)—GPa	20.2
	Mean Young’s modulus (ITZ)—GPa	22.0
	Minimum Young’s modulus (ITZ)—GPa	16.7
	Mean thickness (ITZ)—μm	65
X—glass	Mean Young’s modulus (aggregate)—GPa	86.2
	Mean Young’s modulus (ITZ)—GPa	21.8
	Minimum Young’s modulus (ITZ)—GPa	14.1
	Mean thickness (ITZ)—μm	30
X—plastic	Mean Young’s modulus (aggregate)—GPa	6.5
	Mean Young’s modulus (ITZ)—GPa	19.2
	Minimum Young’s modulus (ITZ)—GPa	12.0
	Mean thickness (ITZ)—μm	50
X—wood	Mean Young’s modulus (aggregate)—GPa	4.7
	Mean Young’s modulus (ITZ)—GPa	19.3
	Minimum Young’s modulus (ITZ)—GPa	15.1
	Mean thickness (ITZ)—μm	60

**Table 3:** Mean of the 24 h water absorption and saturated surface-dry density of RA [13].

Type of RA	Fraction	24-Hour Water Absorption	Saturated-Surface-Dry Density (kg/m^3^)
Concrete waste	FRA	9.5%	2300
	CRA	4.9%	2442
Masonry and concrete waste	FRA	9.3%	2292
	CRA	7.2%	2332
CDW waste	FRA	8.0%	2399
	CRA	5.0%	2399

**Table 4:** Types of CRA and maximum recommended incorporation ratios of EN206 [41].

Type of Coarse RA	Class of Constituents	Exposure Class			
		X0	XC1, XC2	XC3, XC4, XF1, XA1, XD1	All Others
Type A	R_c_90	50%	30%	30%	0%
	R_c_ + R_u_95				
	R_b_10				
	R_a_1				
	X + R_g_1				
	FL_2_				
Type B	R_c_90	50%	20%	0%	0%
	R_c_ + R_u_95				
	R_b_10				
	R_a_1				
	X + R_g_1				
	FL_2_				

## Data Availability

All data presented in this paper may be consulted in the cited references.

## List of Acronyms

CDW	construction and demolition waste
CRA	coarse recycled aggregates
EFTA	European Free Trade Association
EU	European Union
FL	constituent of a recycled aggregate—floating materials
FRA	fines recycled aggregates
ITZ	Interfacial transition zone between aggregate and binder paste
NA	coarse natural aggregates
RA	recycled aggregates
R_a_	constituent of a recycled aggregate—bituminous materials
R_b_	constituent of a recycled aggregate—clay masonry, calcium-silicate masonry, aerated non-floating concrete
R_c_	constituent of a recycled aggregate—concrete and mortar
R_g_	constituent of a recycled aggregate—glass
R_u_	constituent of a recycled aggregate—unbound stone
UEPG	European Aggregates Association
X	constituent of a recycled aggregate—other materials (clay, soils metals, non-floating wood, plastic, gypsum-based and rubber)
